# Physiological Chemistry

**Published:** 1896-08

**Authors:** John A. Miller

**Affiliations:** Professor of Chemistry, Niagara University


### Progress in Medical Science.

### PHYSIOLOGICAL CHEMISTRY.

Reported by JOHN A. MILLER, Ph. D.,
Professor of Chemistry, Niagara University.

### IRON IN FOOD.

RALPH STOCKMAN (*J. Physiol.*, 1895 ; *J. Chem. Soc.*, 1896). The ordinary daily diet was found to contain about 9 or 10 milligrams of iron ; but in chlorotic people, who take but little food, the amount was about 3 milligrams. The following articles of diet were also examined : Milk, from 2 to 4.3 milligrams per liter ; oatmeal, 3.5 milligrams per 100 grams (dried) ; bread, from 0.6 to 0.85 milligrams per 100 grams (dried) ; yellow ox marrow, 2.5 to 4 milligrams per 100 grams (dried) ; red calf marrow, 7.6 to 8.7 milligrams per 100 grams (dried) ; beefsteak, 3.9 milligrams per 100 grams (dried).

---

FORMATION OF BLOOD FROM INORGANIC IRON.

Joseph A. Kunkel (*Pflüger's Archiv.*, 1895 ; *J. Chem. Soc.*, 1896). As a contribution to the disputed question whether inorganic iron is absorbed and contributes to blood (hemoglobin) formation, experiments were made on two puppies ; one received milk, in which the amount of iron was ascertained, the other the same food plus iron. The milk contained about 1 milligram of metallic iron (1.4 milligrams $F_2 O_3$) per liter. The extra iron given daily was 30 drops of liquid ferri albuminat of the German Pharmacopeia (=4.4 milligrams of iron). During life, the blood was examined at weekly intervals. After death other organs were submitted to analysis. A greater amount of iron was found in the dog to which iron had been administered.

---

TOXIC SUBSTANCE FROM THE SUPRA-RENAL CAPSULES.

D. Gourfein (*Compt. Rend.*, 1895 ; *J. Chem. Soc.*, 1896). The glycerine extract of the supra-renal capsules contains proteids which are precipitated by alcohol and have little or no toxic effect, together with substances which are not precipitated by alcohol and are highly toxic. As the latter are not decomposed by heat, the capsules can be extracted with hot water, the solution precipitated by alcohol and the clear liquid evaporated on a water bath. The product when injected subcutaneously causes death in a short time and seems to act on the central nervous system. The proportion of the poison in the supra-renal capsules is variable, but no similar effects are produced by extracts of the spleen or the muscle of the same animals treated in the same way.

---

SUCCUS ENTERICUS OF SHEEP.

Fritz Pregl (*Pflüger's Archiv.*, 1895 ; *J. Chem. Soc.*, 1896). The present experiments were made on a lamb. The juice was collected by a modification of the Thiry-Vella method. It is a mucous, strongly alkaline fluid, which is rich in proteid and tends, like pancreatic juice, to set into a jelly spontaneously ; its specific gravity is about 1,014. It contains about 0.2 per cent. of urea.

The following analysis is given in parts per 1,000 : Sodium carbonate, 3.69 ; albumin and globulin, 18.09 ; albumoses and mucin, 1.27 ; urea, 2.29 ; other organic substances, 3.31 ; ash, 1.27 ; water, 970.05. On proteids, cellulose, pentoses and fats it has no digestive action, but it converts starch and glycogen into dextrose with intermediate dextrins ; it inverts cane sugar and maltose, but not lactose.

---

### ELIMINATION OF CALCIUM COMPOUNDS IN RACHITIS.

WILLIAM OECHSNER DE CONINCK (*Compt. Rend.*, 1895 ; *J. Chem., Soc.*, 1896). In cases of rachitis the quantity of calcium in the urine gradually diminishes in the same manner as the quantity of magnesium. When elimination of magnesium is small, however, that of calcium is relatively high, and this result has led the author to conclude that in rachitis the calcium of the osseous system is partially replaced by magnesium. Chabrie has arrived at a similar conclusion with respect to osteomalagia.

---

### RESPIRATORY METABOLISM.

W. FILEHNE and H. KIONKA (*Pflüger's Archiv.*, 1892 ; *J. Chem. Soc.*, 1896). Geppert and Zuntz (*ibid.*, 42) have shown that during muscular work, in spite of the increased respiratory exchange, the blood gases remain practically unaltered. They consider that this constancy is regulated by nervous action, and one of the questions investigated in the present research is the discovery of the nerve channels and the results of dividing them. Rabbits and dogs were used ; muscles were thrown into tetanus by electrical stimulation and the expired air and blood gases analysed by Zuntz's methods. Observations are also recorded on body weight and temperature.

The result of cutting the nerves of the tetanised muscles is that muscular work caused a fall in the amount of oxygen in the aortic blood ; there is also a small diminution in the amount of carbon dioxide. But when the vagi are also divided there is a marked rise in the latter gas and a fall in the amount of oxygen ; the vagi are supposed to be the chief nervous channels in question, experiments leading to the conclusions that it is on the vagal terminations in the lungs that the venous blood specially acts and thus secondarily on the respiratory center ; and further, that the diminution of oxygen is a more important factor in exciting dyspneic symptoms than increase of carbon dioxide. On the other hand, it appears to be the increase of carbon dioxide in the muscular substance which excites the sensory nerves of muscle and so reflexly influences the respiratory mechanism.

The blood gases and respiratory activity appear to bear no constant relationship to each other ; thus the animal may breathe when the arterial blood has much oxygen and little carbon dioxide, and the pauses may occur when the opposite condition of the blood gases is present. Fatigue of the respiratory center is also dismissed as an explanation of the phenomena ; the real explanation is left an unsettled problem.

---

### ACTION OF SALTS ON THE GASTRIC DIGESTION OF FIBRIN AND OF ACIDS ON THE SALINE DIGESTION OF FIBRIN.

A. Dastre (*Compt. Rend.*, *Soc. Biol.*, 1894 ; *J. Chem. Soc.*, 1896). The proteolytic action of acidified pepsin is prevented by concentrated salts, like sodium or ammonium chloride ; the proteolytic action of concentrated saline solutions is prevented by acidification.

---

### OXIDISING POWER OF THE BLOOD.

J. E. Abelous and G. Biarnès (*Compt. Rend.*, *Soc. Biol.*, 1894 ; *J. Chem. Soc.*, 1896). Salicylaldehyde is not oxidised to salicylic acid by the air or by distilled water or physiological saline solution at 37° C. The acid is, however, formed when defibrinated blood or serum is added at this temperature, although this does not occur at the temperature of the air. The amount of oxidation varies with the blood of different animals and is attributed to a specific ferment, which is destroyed by boiling. Certain organs (testes, thyroid, adrenals, thymus, kidney, liver, lung and spleen) possess the same power, which is lost when vitality is destroyed ; it is not shown by muscles, brain or pancreas.

---

### ACETONURIA.

J. H. Abram (*J. Pathol. and Bacteriol.*, 1896 ; *J. Chem. Soc.*, 1896). The present experiments confirm Becker's original statement that acetonuria follows anesthesia in two-thirds of the cases, the anesthetic used making no difference ; if acetonuria is present before, anesthesia increases it. The probable source of acetone is proteid destruction. The practical outcome is that, except in cases of urgency, anesthetics should not be administered to diabetic patients.

---

### EHRLICH'S DIAZO REACTION.

R. T. Hewlett (*Brit. Med. J.*, 1896; *J. Chem. Soc.*, 1896). Several modifications have been proposed in the original method of testing with sulphanilic acid and sodium nitrite. The reaction is invariably given by the urine in typhoid fever, although occasionally it is seen in other diseases also.

Attention is drawn to the fact that the test solutions must be freshly prepared before using.

The nature of the substance in the urine that gives the reaction is uncertain, and of a large number of materials examined morphine was the only one which gives a similar red reaction, but no green precipitate forms on standing. One part of morphine in 10,000 of water gives the test.

---

### PASSAGE OF SODIUM IODIDE FROM THE BLOOD TO THE LYMPH.

L. B. Mendel (*J. Physiol.*, 19; *J. Chem. Soc.*, 1896). This is a contribution to the vexed question of lymph formation. The author supports Heidenhain's view that physical processes will not explain lymph formation. Sugar and sodium chloride occur in greater amount in lymph than in blood, and the same is true for sodium iodide after injection into the blood stream.

These facts are considered to bear out the doctrine of lymph secretion.

---

### INTESTINAL ABSORPTION OF PEPTONE.

E. H. Reid (*J. Physiol.*, 1896; *J. Chem. Soc.*, 1896). Experiments were made on dogs, loops of the intestine being measured off and tied by tapes; into these recently exposed loops the peptone was injected; parallel experiments were always conducted on two loops. After a quarter of an hour the dogs were killed, the loops were removed and their contents examined. A table of six experiments is given, showing an absorption of from 40 to 70 per cent. of the peptone introduced; the difference between the two loops was never more than 5 per cent.

---

### UREA IN ANIMAL ORGANS.

B. Schöndorff (*Pflüger's Archiv.*, 1895; *J. Chem. Soc.*, 1896). By means of the method previously described by the author he finds in the organs of a dog, with the exception of the muscles, heart and kidneys, urea in quantity corresponding with that in the blood.

The muscles contain urea. This is a direct contradiction of the work of previous physiologists ; it is present in too large an amount to be accounted for by the blood in the muscles.

Urea is a constituent of the red corpuscles, and is indeed about equally divided between blood corpuscles and blood serum.

